# Supplementary material for: Protective Effects of N-Acetylcysteine on Lipopolysaccharide-Induced Respiratory Inflammation and Oxidative Stress
Source: Antioxidants (Basel). 2022 Apr 29;11(5):879. doi: 10.3390/antiox11050879 (PMC9137500; doi:10.3390/antiox11050879)
Supplement: Supplementary file 1 [file antioxidants-11-00879-s001.zip › antioxidants-1624246-supplementary.pdf]

Table S1. Determination of CAT, SOD, MPO and MDA

Indicators	Description about methods
Catalase (CAT)	The kit uses the ammonium molybdate method, that is, the reaction of CAT decomposing H_2O_2 can be quickly terminated by ammonium molybdate, and the remaining H_2O_2 reacts with ammonium molybdate to form a pale-yellow complex, whose OD value can be measured at 405nm to calculate CAT activity. Before determination, it is necessary to dilute 10% tissue with physiological PBS to different concentrations for pre-experiment, the proportional concentration of homogenate was selected for formal experiment. One unit of CAT activity was defined as the amount of enzyme decomposing 1 μmol of H_2O_2 in 1 s. The final result was normalized by protein concentration (mgprot/mL) and the unit of the final result was U/mg prot.
Superoxide Dismutase (SOD)	This detection adopts WST reaction system that contains xanthine; xanthine oxidase produces the superoxide anion free radical ($O_2^{\cdot-}$). WST can react with the superoxide anion in the tissue to produce water-soluble dye WST formazan, and this process can be inhibited by SOD. Before using this kit to test samples, individual samples should be selected and diluted to different concentrations for pre-experiment. A dilution ratio of 40%-60% SOD inhibition rate should be selected for formal

	test. After each sample was tested according to the kit instruction, the OD value was read at 450nm wavelength. In this reaction system, when the SOD inhibition rate reaches 50%, the amount of enzyme correspond to one SOD activity unit (U). The limit of detection is 0.5 U/mL. The final result was normalized by protein concentration (mg prot/mL) and the unit of the final result was U/mg prot.
Myeloperoxidase MPO	Myeloperoxidase (MPO) is a heme-containing enzyme that catalyzes the hydrogen peroxidase-mediated oxidation of halide ions to hypohalous acid. In the MPO assay protocol, myeloperoxidase produces HClO from H2O2 and Cl-. The HClO reacts with taurine to generate the taurine chloramine, which subsequently reacts with the DTNB probe to eliminate color (absorbance at 412 nm). The absorbance is inversely proportional to the amount of MPO enzyme. MPO activities were measured by OD value differences. The final result was normalized by protein concentration (/mL) and the unit of the final result was U/gram.
Malondialdehyde MDA	Lipid peroxidation products (thiobarbituric acid-reactive substances) can be combined with thiobarbituric acid (TBA) to form a red product with a maximum absorption peak at 532nm. After finishing the reaction, measure absorbance immediately on

	a microplate reader at OD 532nm for colorimetric assay. The variable coefficient (CV)= 1.5%. Lipid peroxidation was expressed as mmol of MDA per milligram of protein.
